# “*I would much rather hear the truth so I can be prepared*”: Virtual peer support, health access, and information-seeking after hysteroscopy referral

**DOI:** 10.1177/17455057261484349

**Published:** 2026-08-31

**Authors:** Susanne Katharina Cromme, Richard Harrison, Katherine A. Finlay

**Affiliations:** 1 School of Psychology and Clinical Language Sciences, Department of Psychology, 150627 University of Reading, Harry Pitt Building, Earley, Reading RG6 6ET, Reading, United Kingdom of Great Britain and Northern Ireland

**Keywords:** patient information, women’s health, virtual peer support, digital health access, informed consent, patient safety, behaviour change technique taxonomy, theoretical domains framework

## Abstract

**Background:**

Deficiencies in patient information undermine informed consent and patient safety in gynaecological care. When clinical communication fails, women turn to digital peer communities, directly affecting health access and decision-making. Hysteroscopy procedures are at heightened risk of this failure: pre-procedural information is routinely unstandardised, and anxiety affects up to 80% of patients, impairing attendance and completion.

**Objectives:**

This study investigated women’s information needs following hysteroscopy referral, using naturally occurring online data to identify behavioural targets to improve care and patient safety.

**Design:**

A framework analysis of naturalistic online forum data.

**Methods:**

Data were purposively sampled from threads containing “hysteroscopy” in the title, posted on Mumsnet.com. A total of 5,671 posts were extracted from 343 discussion threads and analysed inductively using Framework Analysis. Themes were mapped to the Theoretical Domains Framework, with key behaviour change techniques identified using the Behaviour Change Technique Taxonomy.

**Results:**

Women were unable to access clear, foundational information about hysteroscopy. Given this deficit, four critical domains of basic information need were identified: 1) navigating procedural processes, 2) anticipated pain trajectories, 3) managing the recovery phase, and 4) diagnostic outcomes and aftercare. Repeated information-seeking queries suggest an 'information neglect continuum': virtual peer support fulfils a compensatory role where formal communication was perceived as insufficient. Behavioural mapping identified actionable intervention points to strengthen patient instruction, repositioning informational integrity as a core component of clinical quality, not an adjunct to it.

**Conclusions:**

Routine hysteroscopy communication fails to provide the structured guidance women need to consent to and recover from the procedure. Self-initiated information-seeking within a virtual support network revealed deficits not captured by conventional service evaluation. This study establishes how digital social support mitigates clinical information failures and confirms which information components patients prioritise. These represent priority intervention targets with direct implications for informed consent, procedural attendance and patient safety.

## Plain Language Summary

Hysteroscopy is a common gynaecological procedure used to examine the inside of the womb. It is performed to investigate symptoms such as heavy or irregular bleeding, fibroids, or fertility problems, and involves passing a small camera through the cervix while the patient is awake. Despite how frequently it is carried out, many women arrive at their appointment with very little understanding of what it involves, how painful it might be, or what to expect afterwards. This is not because the information does not exist; it is because it is not reliably provided through clinical sources. This study set out to understand what women actually want to know about hysteroscopy. To do this, we analysed over five thousand posts from 343 discussion threads on Mumsnet, the UK's largest online forum for women, spanning seven years. Rather than asking women directly in an interview or survey, we looked at the questions they asked unprompted, in their own words, in real time. This approach captures what women genuinely need to know, without the filters of a formal research setting. Four clear themes emerged. Women wanted to understand why they had been referred and what the procedure was actually for. They wanted honest, specific information about pain: data on how much pain to expect, for how long, and why it varies so much between women. They wanted to know what recovery would look like in practical terms: when they could drive, go back to work, exercise, have sex, or start trying to conceive. Also, they wanted to know what happened after the hysteroscopy: when results would arrive, what they would mean, and who to contact if something felt wrong. These are not complex or unusual questions. They are the basic questions any informed patient should be able to answer before consenting to a procedure. That thousands of women are turning to strangers on an internet forum to get this information is a significant finding. It tells us that this ‘information neglect continuum’ is not individual or occasional. It is systemic and predictable. Crucially, Mumsnet is not functioning simply as an information source. Women in these threads are offering each other reassurance, sharing their experiences to help others calibrate their expectations, and collectively constructing the care guidance they were never given. This is a form of virtual peer support, and it has real consequences for health. Women's decisions about whether to attend their appointment, whether to accept or decline procedures offered during hysteroscopy, and how to manage their recovery are being shaped by what they read in these threads, not by their clinical team. This matters for how we design patient information and digital health tools. It also matters as a warning: when clinical services routinely fail to communicate, women do not simply go without. They build their own support systems which often rely on non-evidenced information. That the onus falls on the women to self-educate is the problem this research asks us to take seriously.

## Introduction

Hysteroscopy is the gold-standard procedure for visualising the uterine cavity and is commonly used in the investigation and management of common gynaecological concerns such as post-menopausal bleeding, heavy menstrual bleeding (HMB), fibroids and reduced fertility.^1^ The procedure involves the insertion of a thin, telescope-like instrument through the cervical canal to examine and, where required, treat intrauterine pathology.^2^ Recent system-wide pressures have intensified the challenges surrounding hysteroscopic care.

Gynaecology waiting lists have risen by over 60% compared with pre-pandemic levels, leaving more than 750,000 women awaiting treatment.^3^ Prolonged delays are associated with worsening presentations, reflected in a 33% rise in emergency admissions in England between 2021 and 2024.^4,5^ Concurrent increases in outpatient non-attendance^6^ warn of declining engagement and highlight the need to actively improve modifiable determinants such as informational preparedness.

The quality of pre-appointment information is associated with improved attendance rates and procedural success, whereas inadequate or delayed explanations regarding logistics, processes, or purpose are explicitly implicated in poorer outcomes and satisfaction rates.^7^ Inadequate information also risks causing confusion and anxiety,^8^ particularly in hysteroscopy, where fear of pain, prior negative experiences and uncertainty underpin the association between pre-procedural anxiety and informational need.^9^ Anxiety affects up to 80% of outpatient hysteroscopy patients^9–11^ and impedes both attendance, pain perception and procedural completion.^12–15^ These findings emphasise the critical role of comprehensive information in supporting patient engagement and scaffolding procedural success.

Meaningful shared decision-making and informed consent, in line with RCOG’s Green-top Guideline No. 59,^16^ require accessible, accurate information about condition and management options.^17,18^ Improving the accessibility and the quality of women’s health information is recognised as a global priority, underscored by the World Health Organisation and the World Health Assembly.^19,20^ However, despite explicit NICE guidance on the need for patient-centred, comprehensive pre-procedural and clinical information,^21^ existing written materials across NHS Trusts remain unstandardised.^22,23^ Indeed, they have been criticised by medical professionals as “inaccurate, inconsistent, and confusing”.^24^ The Renewed Women’s Health Strategy for England^25^ explicitly identifies improving access to clear, timely information as a central and achievable priority. As such, understanding what information patients find useful and how to deliver it accessibly remains essential for improving comprehension, engagement and the patient experience.

The mismatch between the information patients require and what they receive often drives women to seek guidance beyond formal clinical sources. Approximately 57% of gynaecology patients use social media to seek women’s health information, and 58.6% report that social media supports their health-related decision-making.^26^ This is a pattern that positions digital platforms not simply as supplementary sources, but as functional substitutes for the social support and guidance that clinical encounters fail to deliver. Empirical analyses of online platforms, including studies of IUD insertion and removal, have previously provided valuable insight into women’s health-seeking behaviours and procedural experiences; however, women’s informational needs at the point of referral have largely been overlooked.^27–30^ Online forums provide a valuable naturalistic dataset in this context, capturing unprompted questions that reveal unmet informational needs not easily identified through traditional qualitative research design. Critically, these forums also constitute sites of virtual social support: spaces where women establish trust, share experiential knowledge, and collectively construct the care guidance they have not received. This has direct consequences for health access, procedural engagement, and informed consent. This study seeks to analyse forum-users’ questions, concerns and information needs across the hysteroscopy care pathway. Ultimately, by mapping the resulting themes to behavioural frameworks, the findings are translated into potential opportunities for service improvement, at the individual and system level.

## Methods

### Design

This qualitative study analysed publicly available online forum discussions using framework analysis to examine women’s information needs following hysteroscopy referral.

### Setting

Data were extracted from Mumsnet.com, a large UK-based interactive forum where health topics are frequently discussed.^31^ This platform was selected as a purposeful data source on the basis of its UK-wide reach, large and active userbase, and frequent publicly accessible discussions on women’s health topics. With 33.1 million monthly visits and approximately 700,000 posts per month, it represents one of the largest repositories of naturally occurring women’s health discourse in the UK and attracts a diverse user base, with 62.2% aged 25–54 and 52.61% identifying as female.^32^ The platform has been widely utilised across disciplines as a source of women’s perspectives on health, sociocultural, and political issues,^30,33–40^ and critically, captures experiences from across the UK, overcoming the geographical constraints of studies limited to single NHS Trusts. A characteristic of the platform is that individual demographic data are not available for forum contributors. Nevertheless, the dataset comprised over 5,600 posts reflecting organically diverse queries encompassing varying referral symptoms, menopausal status, parity, and comorbidities. In line with established guidance for internet-mediated research,^41^ the forum was treated as a public space; no interaction with users occurred and all quotations were anonymised.

### Data extraction

Data were purposively sampled from publicly available Mumsnet posts, commencing from the date of the adoption of NICE guideline NG88^42^ on outpatient hysteroscopy (4 March 2018) to 31 December 2025. Ethical approval was obtained on 13.06.2023 from the University of Reading School of Psychology Ethics Research Committee (2023-096-KF). and Mumsnet administrator permission was secured prior to extraction. Data were included for analysis if posts appeared within discussion threads containing the term “hysteroscopy” in the thread title. Threads not meeting this criterion were excluded, as an initial broad search demonstrated that title-based restriction was necessary to minimise irrelevant content. All posts within eligible threads were retained for analysis regardless of individual post content. These threads were extracted using R Statistical Software (V4.1.2)^43^ with the rvest,^44^ dplyr^^45^^ and tidyr^46^ packages. Data extracted comprised post content, usernames and post dates only. Beyond usernames, the platform does not display personally identifiable information, and no demographic, locational, or other identifying data were collected. Quotations are presented verbatim with typographical corrections. Following Mumsnet’s research protocol, usernames were omitted and no identifying metadata was retained, re-identification risk through search engines is considered minimal. Informed consent from individual participants was not required and was waived by University of Reading School of Psychology Ethics Committee on the grounds that the data analysed comprised publicly accessible online forum posts, no interaction with users occurred, and no personally identifiable information was collected or retained.

### Data analysis

343 discussion threads containing 5,671 posts were scraped using R. Posts were reviewed for relevance and all were retained for analysis. The qualitative textual data derived from forum posts were uploaded to MAXQDA^47^ and analysed using framework analysis (FA).^48^ This research is reported according to the consolidated criteria for reporting qualitative research (COREQ).^49^ See supplementary material 1.

### Analytical approach

Data were analysed using framework analysis as it is a systematic approach well suited to applied qualitative health research and the analysis of large, heterogeneous textual datasets such as online forum discussions.^48,50^ An inductive approach^51^ was adopted first to identify the information women sought following referral for hysteroscopy.

All forum posts were read repeatedly to support familiarisation. Initial coding was conducted inductively by the first author, capturing information needs, uncertainties and experiential concerns expressed across threads. Codes were iteratively refined and grouped into themes and subthemes, which were subsequently organised into higher-order domains reflecting different stages of the hysteroscopy pathway. Data were charted into a framework matrix^50^ to enable comparison across threads while retaining links to illustrative quotations. Theme development, refinement and naming were discussed and agreed through regular analytic meetings involving all members of the multidisciplinary research team. A reflexive journal was kept throughout the analytical process and preparation of the manuscript. Thematic development focused on the coherence of the analysis, not on reaching a point of data saturation.^52^ Quotations are presented verbatim in Table 1, with typographical and grammatical errors amended to enhance readability.^8,53^ In keeping with the epistemological basis of framework analysis, themes are not quantified by frequency of occurrence; analytic significance is determined by relevance to the research question and depth of meaning rather than prevalence within the dataset, consistent with established qualitative and applied policy research methodology.^50,53,54^

**Table 1.** Framework matrix table: Domains, themes and sub-themes of women’s online information seeking after hysteroscopy referral.

Domain	Theme	Subtheme	Quote	ID
Navigating Procedural Processes	Access to care	Length of waiting list	*I just wondered how long after consultation would I have to wait to have hysteroscopy? Would it be another year or months? Or weeks?*	28
			*I got an appointment really quickly, and I am now freaking out, as the NHS doesn’t normally move very fast*.	134
		Private Healthcare Cost	*Anyone ever paid for private hysteroscopy (camera put in womb) NHS appointment? I’ve been waiting months for it to have just been cancelled again*. *Should have also said, how much was it?*	76
		Unfamiliarity with pre-operative appointments	*Have had my pre-op and have now been referred to the anaesthetist*. *I have had similar surgery before under a GA [General Anaesthetic] and never had to see the anaesthetist*. *Does anyone know why this would happen?*	441
			*Next step is a hysteroscopy - I have my pre-assessment today and have no idea what to expect! Anyone else had this done?*	475
		Barriers to procedure completion	*Has anyone had this done recently with the NHS? What will happen if I’m on my period? Can this still be done? The date they have given me is in September and I’m due my period the day before operation*. *I’m scared in case they will cancel it and I will have to wait even longer for this operation to be done*. *Been TTC [trying to conceive] for 3 1/2 years*.	102
			*There’s absolutely nothing in the leaflet/on the confirmation letter about pregnancy or contraception, crazy*. *Plus, the referring consultant said nothing either*. *I’ll be cd23 [cycle day 23] so it will be too early for a pregnancy test*.	784
			*Will I need to stop my HRT [Hormone Replacement Therapy]? I feel worried*.	787
	Therapeutic justification	Rationale for hysteroscopy referral	*Can I ask if any of you had a D&C [Dilation & Curettage] as part of it? I don’t really understand what that bit is for*. *Not sure what they’re looking for on the hysteroscopy either as I’ve had a transvaginal ultrasound and nothing was found (two years ago- and still having the same irregular bleeding, between periods, etc and am under gynae [gynaecological care]).*	220
			*I received an appointment for the end of September for the procedure*. *Apparently, it’s just a routine hysteroscopy and I was never on the 2-week pathway*. *I thought the biopsy was to rule out abnormal/cancer cells*. *What circumstances would a postmenopausal women require a hysteroscopy? It doesn’t make sense to me*…	3
			*I wish someone had been able to tell me that there was something a bit less unpleasant than a hysteroscopy that might be suggested - surely my GP should have known?*	372
		Therapeutic grounds for Mirena Coil	*Only thing I was surprised by and would have liked more time to consider was the other of a Mirena coil, I’m 53 for [expletive] sake*. *I said no and having researched since I happy I made the right decision but if it’s worth looking into not trying to make a decision, legs akimbo*.	1589
			*Should I just not bother to have the hysteroscopy and biopsy if I don’t want the coil?*	793
			*I have an appointment for the above procedure, Dr wants to fit a Mirena at the same time*. *Made me sign a consent form for both, but no real discussion as to benefits of Mirena*. *I’m peri-menopausal with heavy bleeding*. *Any experiences? Should I say yes, wait, no, what about HRT instead or as well?*	284
	Preparation for Operation	Logistical preparation	*I’m presuming I bring pads and stuff in case I bleed a little*. *I’ll have money to get taxi home*. *Anything else I should think about?*	7
			*Can you have a friend or family member with you for this procedure?*	701
			*Anyone who has had it done, should I take someone with me? Not sure about driving afterwards or how I will feel?*	106
		Anxiety Management Options	*Is there anything I can do to make the fear and anxiety go away?*	3
			*GA isn’t an option unless I delay this appointment further*. *Did they give you something to sedate/calm you?*	620
			*I took paracetamol in advance of the hysteroscopy, but that was all*. *I got a GP to prescribe Valium for the brachytherapy - would that help for the hysteroscopy?*	85
	Procedural Processes	Step-by-step description	*If I need a biopsy on anything, would they do that there and then or would I have to go through it all again?*	70
			*So, [I] have been referred to the hysteroscopy clinic which apparently, I will be awake for, and they will use some water to open everything up?? Does anyone know any more about this? […]do they do a biopsy to be sent off at the same time as the hysteroscopy - or does the hysteroscopy replace that completely?*	58
			*Just wondering how they can take samples/do hysteroscopy with a Mirena in situ*. *Do they take it out and put it back?*	100
			*On the letter no explanation [of] what they [are] actually going to do*. *Are they going to take biopsy? Or they hoping to do something re cyst -fibroids*. *Can I drive after the treatment?*	824
		Procedural duration	*Did you have biopsies too? Do they take long to do?*	37
			*Has anyone had polyp removed? How long did it take?*	39
			*Was it a whole day case?*	244
Anticipated Pain Trajectories	Procedural Pain	Likelihood of pain occurrence	*Anyone else wondering [why] there is a big disparity between the level of pain or discomfort felt by different women during a hysteroscopy, and there’s no telling how it will affect you before you have it*.	114
			*I’ve read that if a woman has had pain during pap smears, that hysteroscopies without GA are horrific?*	14
			*I have just realised I’m going to be on my period during my laparoscopy and hysteroscopy! Really worried this is going to be even more painful than it should be already*. *Plus, not the nicest for the surgeons?!I can’t rearrange as they don’t have any other dates to offer me*. *Does anyone have any advice please??*	23
			*Was it the speculum that hurt, the method used by the doctor or personal medical issues? I didn’t have any polyps removed so I have no experience of that, but what I did have was a Hysteroscopy with a biopsy without ever having a vaginal birth which some have warned against*.	446
			*Women’s health needs to stop relying on anecdotes or 84-year-old friends or GPs not really knowing “but in their experience…”. Women deserve stats*. *Frequently refreshed stats*. *Free from vested interests*.	207
			*I also have a tilted womb*. *Maybe it's why it hurt me so much*.	171
		Severity of procedural pain	*I am due to have a hysteroscopy and, needless to say, I am not exactly looking forward to this procedure*. *The letter sent from my local NHS hospital talks of ‘pain and discomfort’*. *Localised pain killer injection into the cervix*. *I am assured that ‘the procedure can be stopped at any time’*. *Please can anyone tell me of their experiences of this procedure? Please don’t sugar coat it if it wasn’t very pleasant*. *I would much rather hear the truth so I can be prepared*.	267
			*Just how much pain should I expect?*	314
			*Can I highjack slightly and ask if any of you have also had an endometrial biopsy? How the pain levels compare?*	105
			*But what is the standard of pain for “tolerate”? Not passing out? Excruciating pain but resolves quickly? Didn’t punch the consultant in the face? I was probably classed as “tolerating” mine despite having to be physically pinned down in total agony*.	46
		Duration of procedural pain	*I really don’t know how good my pain threshold is to be honest and it’s difficult to know when you don’t know how painful you will find it*. *Do you remember how long you were in pain for when they did the procedure? She said it’s about 1-2 minutes*.	84
	Strategies for Pain Management	Availability of pain relief options	*I am not going through it without anaesthetic - for several reasons*. *I asked the gynae secretary why conscious sedation isn’t offered as standard as it is in other clinics such as colonoscopy or endoscopy, but she didn’t know*. *Anyone know why? It seems bonkers that it's either outpatient (possibly with local - although from what I’ve read this isn’t effective) or a GA, with nothing in between*. *I was just curious as to why I’ve had to push for a GA and why it’s that or nothing - it seems really extreme*.	4
			*If you have a procedure with effects so horrendous that women routinely buckle after having it, why would you just persist with the same method? Why is women’s pain baked in as an expectation?*	135
		Administration of pain relief	*For those of you who have had GA for this procedure, is it just a quick injection?*	156
			*It says I may get a local if they have to remove anything but then they will try it without first*. *Did you have the local before anything was done or did you have it part way through?*	272
			*Also, does a local anaesthetic for this procedure mean a spinal or is it literally a local anaesthetic near the site?*	623
		Effectiveness of analgesia	*Were you effectively asleep or did you know what was going on?*	84
			*Did it hurt you with just the local anaesthetic?*	39
			*I will be offered gas and air or injection this time*. *Has anyone had gas and air, and did it help?*	209
			*Was the epidural or spinal completely paralyzing you?*	94
			*If you had one with a local, what was your experience like? I am out of my mind terrified about it*. *Even the thought of an injection into your cervix sounds bloody horrific*. *And I think that will only numb my cervix, so would I still feel the poking about in my womb?*	996
			*I have never been sedated*. *Can anyone tell me what the experience will be like? Will I still feel the pain? What will recovery be like?*	31
	Post-Procedural Pain	Severity of post-procedural pain	*I am extremely nervous due to many factors and just wondered how others found the whole process? I am worried that I will be quite sore afterwards as I am quite sensitive to pain in that department*.	190
			*Just looking for anyone to share their experiences*. *I had a long recovery after my c section and had ongoing abdominal for approx*. *a year and a half after so very worried about the pain following the procedure*. *Could anyone share their experience with me, I have a 4- and 2-year-old but partner will be taking time off to help*.	1126
		Duration of post-procedural pain	*Just wondering if your pain settled down, and if so, how long did it take?*	127
Managing the Recovery Phase	Restoration of Health	Post-procedural symptoms	*What to expect afterwards, how much bleeding is acceptable?*	107
			*I had Hysteroscopy and polyp removal 1 week ago when they also fitted a Mirena*. *I’m having excruciating cramps still and [it] doesn’t seem to be lessening*. *I’ve no idea what is normal for the settling in period!*	127
		When to seek help	*I had a hysteroscopy on Friday*. *Tonight I’ve started bleeding really heavily with lots of cramping and clots*. *I’m currently waiting for a call back from a 111 nurse and I’m stressing out*. *Anyone had any experience with this? I don’t know what to expect*.	1244
			*This last bleeding has been five days now and could perhaps just be a normal, albeit heavy, period returning*. *Just asking wise MNers [Mumsnet Users] who have experienced this, if I need to be contacting my GP, or worrying yet?* *I wouldn’t be normally worrying at all […] I am worried about leaving any concerns to the last minute*.	249
		Expected recovery timeline	*How long after the procedure until you could go home please?*	192
		Activity restrictions during recovery	*How did you find it? And how long did it take you to recover? Scheduled for next week and wondering when I’ll be fit to look after the DC [Dear Children] (4&10 months) and when I can run, swim again?*	651
			*How long do you need to refrain from sex for?*	107
	Reproductive Health Considerations	Impact on menstrual cycle	*I wanted to know how soon your period came after and what it was like?*	937
			*They advised me not to use tampons or a cup until after the first post procedure menstrual period has finished*. *If that makes sense*. *So maybe this current bleeding is the next menstrual period right now, who knows? I think I might ring the GP*.	735
			*I had a hysteroscopy*. *My period is 2 weeks delayed; anyone know if this is normal?*	812
			*Has anyone conceived after having a hysteroscopy and how long did it take? Did it affect your periods?*	813
			*I just had one and hoping to start trying on my next cycle*. *Did it interfere with periods/ovulation and how long until you conceived?*	1597
			*My period came three days ago, I am bleeding very heavily, filling mooncups repeatedly and leaking*. *Does anyone know if this is normal?*	1491
		Guidance for fertility pathway	*Today I underwent my hysteroscopy which they said went very well and everything has now been removed*. *However, no one really gave me any information on where to go from here*. *When is safe to start trying again for a baby? I don’t want to risk in any way me doing something that could end in a miscarriage again*. *I want to start trying as soon as possible, but what is recommended?*	791
			*how soon were you to start fertility treatment and what was the outcome?*	937
			*I have read if I undergo these procedures I need to wait several months before I can have IVF…is this true? Does anyone have any insight?*	823
			*Also, how long did you have to wait to heal before an embryo could be transferred?*	399
Diagnostic Outcome(s) and Aftercare	Communication of Diagnostic Results	Waiting times for biopsy or procedure results	*Obviously, I’m worried and the waiting for results is horrendous*. *Anyone else had this done? How long did you have to wait for biopsy results? Should I ring my consultant? I don’t want to nag them; I know the NHS is creaking under pressure, but I just can’t concentrate on anything else at the moment*. *I know it’s probably nothing and just hormonal - I’m peri menopausal aged 49*. *I need to stay off google!*	89
			*Also, can I ask whether you’ll get any results back immediately after the procedure or do they wait for the biopsy results to tell you that (and how long would that take). Do they tell you anything apart from sending the biopsy off that day?*	7
			*I waited 8 weeks and they just told me sorry they have lost the results, what do I do now?*	28
		Clarity of result communication	*Just wondered if this is common*. *Hysteroscopy in Day unit but when I received results it says insufficient tissue for biopsy*.	58
			*The consultant said I would need a biopsy almost as soon as he began the scan which has really frightened me thinking he seen something terribly wrong right away*. *Anyone had a similar experience? What was the outcome? So frightened*.	86
			*I now feel totally at a loss as to what happened, can a polyp disappear on its own? One of the nurses said it is possible that a polyp can retreat back into the lining of the endometrium which worries me more as does that now mean I’m playing a waiting game for it to reappear? And could it be possible that a polyp is missed whilst having a hysteroscopy?*	1679
			*I had a hysteroscopy a few weeks ago due to multiple polyps*. *These were removed and I received a letter saying there were no abnormal cells*. *I have now received a letter calling me to see the gynaecologist*. *Anyone have any reason why?*	441
	Continuity of Care	Provision of follow-up care	*Did you have follow-up appointments e*.*g*. *another scan or blood tests [after] the operation?*	52
			*And if nothing was found ‘wrong’ and it was put down to being hormonal, was anything offered?*	7
			*Are they just monitoring the lining each year?*	86
			*I’m on the 12-month smear call and unsure if hystero[scopy] cancels the need for a smear but I do keep getting the text and letter reminders about booking for smear which makes me think otherwise*.	91
		Accessibility of post-procedural support contacts	*Were you given an aftercare help number by the hospital where your hysteroscopy was done?*	2
			*Where do I get support for the trauma this incident has caused me?*	32

### Reflexivity and rigour

The research team comprised a psychotherapeutic counsellor (female), health psychologist (female) and women’s health researcher (male), none with personal experience of hysteroscopy. The absence of a hysteroscopy clinician on the research team was a deliberate methodological decision. As the study’s analytical focus is women’s information-seeking behaviour and experiential knowledge expressed in naturalistic peer discourse, interpretation was conducted through a behavioural and psychological lens consistent with the study’s epistemological position. Introducing clinical mediation at the analytical stage risked imposing a normative procedural frame onto data whose value lies in its independence from clinical prompting. A reflexive log documented analytic decisions and assumptions. Rigour was enhanced through iterative coding, team discussion and systematic cross-case comparison. Reporting followed COREQ guidelines^49^ (see Supplementary Material 1).

### Behavioural framework mapping

Following inductive framework analysis, themes and subthemes were mapped post-hoc to the Theoretical Domains Framework (TDF)^55^ to identify behavioural determinants of women’s information needs. Subthemes were assigned to one or more domains where appropriate. Identified TDF domains were then used to select relevant behaviour change techniques (BCTs) from the Behaviour Change Technique Taxonomy v1 (BCTTv1),^56^ indicating potential targets to strengthen patient-centred information provision across the hysteroscopy pathway.

## Results

### Thematic framework analysis

**Figure 1.**
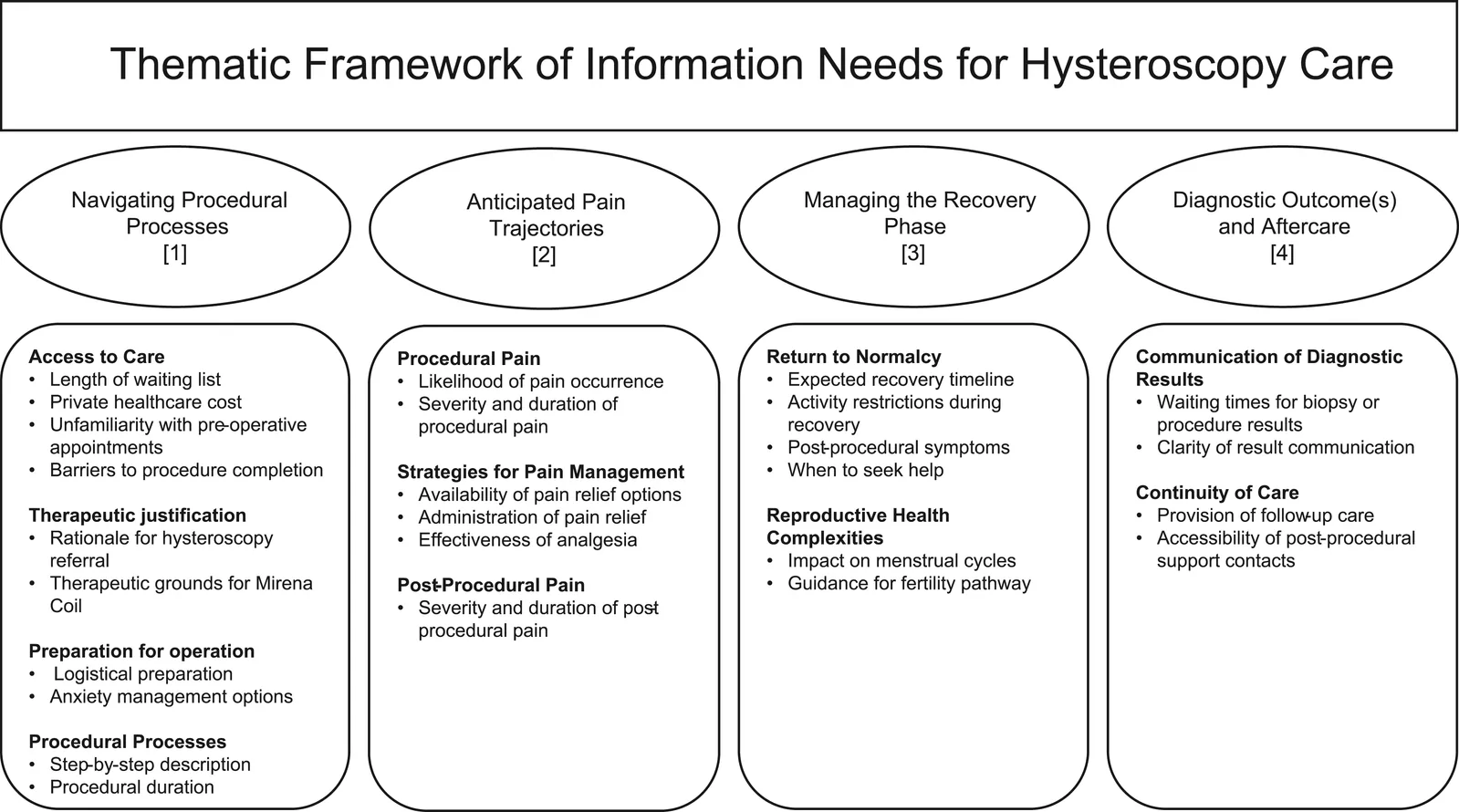
Note: Ovals represent core domains, bold headings represent themes and bullet points represent sub-themes deriving from the framework analysis into hysteroscopy information-seeking.

The analysis identified four core thematic domains (Figure 1): (1) Navigating Procedural Processes, (2) Anticipated Pain Trajectories, (3) Managing the Recovery Phase, and (4) Diagnostic Outcomes and Aftercare.

Each domain (ovals) contains themes reflecting the primary focus of women’s questions about hysteroscopy, with subthemes illustrating the breadth of information sought. Findings indicate consistent needs for anticipatory guidance, procedural explanation and follow-up care.

## Domain 1: Navigating procedural processes

Across this domain, participants’ narratives revealed a continuum of informational absence that begins with uncertainty about when and why care occurs and extends through to how an intervention is prepared for and experienced. Participants’ primary concerns covered: *access to care, therapeutic justification, preparation for operation, and procedural processes*.

### Access to care

Forum-users frequently sought clarity on waiting times, attempting to define what was “normal” and interpret rapid or delayed referrals as markers of urgency or system failure. They compared timelines and experiences to impose an illusion of order on inconsistent scheduling. Questions about pre-operative appointments, menstrual timing, HRT [Hormone Replacement Therapy] and pregnancy reflected efforts to understand administrative or clinical barriers that might delay treatment. Some also explored private costs, calculating alternatives when NHS access faltered. Collectively, these exchanges reveal active attempts to secure procedural clarity within a system experienced as opaque, where access was perceived to depend not only on clinical need but, in part, on a patient’s ability to navigate ambiguous logistics with limited informational support.

### Therapeutic justification

This domain focused on understanding why hysteroscopy was necessary and what it aimed to achieve. Participants’ questions reveal a desire for diagnostic transparency: what the procedure could detect, why it was indicated post-menopause, or whether it was purely routine. In the absence of clear clinical reasoning, many also investigated the therapeutic rationale for interventions offered concurrently, especially the Mirena coil insertion. Participants sought to understand its purpose, benefits, and implications for consent. These exchanges frequently centred on weighing up whether declining a coil might affect their care, or exploring whether alternative, less invasive options were available. Such reflections point to informational gaps about diagnostic intent, benefit and consent.

### Preparation for operation

Participants’ preparatory information seeking was both practical and emotional. They turned to forums to crowdsource essential logistical details: what to bring, whether sanitary pads would be provided, and if accompaniment by partners/important others was allowed. Participants sought to reproduce the guidance a clinical consultation might have offered, by building checklists and coping strategies through peer-generated knowledge within the forum. Importantly, information seeking extended to emotional preparation: users asked others how to manage fear and whether pharmacological intervention could be requested to help to calm nerves prior to the procedure.

### Procedural processes

Participants consistently reported minimal understanding of what would occur during hysteroscopy, requesting step-by-step honest descriptions from peers, asking questions about instruments, the use of water, whether the cervix was dilated, and if biopsies or removals might occur concurrently. This desire for detail reflects a desire to prepare for the experience. Even after the procedure, participants sought clarification by comparing accounts to understand what had occurred, what was normal, and to regain a sense of control.

Taken together, a pervasive informational void at the beginning of the hysteroscopy pathway is exposed. This forced patients to become their own navigators, interpreters, and educators as they struggled to make sense of access barriers, clinical reasoning, emotional preparation and procedural processes.

## Domain 2: Anticipated pain trajectories

This domain represents forum-users’ most intensive and desperate information seeking focus: Pain. Participant queries highlighted their uncertainty about how much, why, and for how long procedural pain would occur, and what options exist to prevent it. Queries about *procedural pain, strategies for pain management, and post-procedural pain* reveal that pain is not simply a physical outcome, but that it must be considered within the context of patient communication and emotion.

### Procedural pain

Information-seeking around procedural pain reflected an urgent effort to predict and quantify potential discomfort. Forum-users requested descriptions of pain intensity, duration and key contributing factors, using experiential comparisons to assess personal risk. They called for statistical or clinical evidence to replace inconsistent reassurance and attempted to map pain likelihood across variables such as clinician technique, menstrual phase, uterine position and prior smear or biopsy experiences. Honest first-hand accounts were sought to calibrate realistic expectations and tolerance thresholds. This reflects a demand for evidence-based communication: participants wanted pain framed as a foreseeable, measurable outcome rather than an unknowable risk.

### Strategies for pain management

Information-seeking around pain management reflected both practical and critical inquiry. Forum-users sought clarity on available pain relief options and their administration, attempting to map a spectrum that in practice appeared binary: between unmedicated outpatient procedures and general anaesthesia. To identify viable strategies, women asked questions about the experience and efficacy of offered pain-relief, and the advisability of over-the-counter analgesics. Yet such questions also carried critique: Forum-users interrogated why intermediate options available in other outpatient procedures, like conscious sedation, were absent for hysteroscopy. Forum-users experienced such limitations in pain management as being representative of a general gendered indifference to female pain.

### Post-procedural pain

To align recovery expectations with lived experience, participants sought benchmarks for what constituted ‘normal’ post-procedural discomfort, asking others how long pain persisted, and whether soreness would affect caregiving or daily functioning. The emotional dimension of post-procedural uncertainty, particularly among those with prior difficult surgical recoveries, transformed physical pain into a wider concern about bodily resilience and preparedness. Physical pain extended into emotional and social components, shaping recovery expectations and support needs. Discussions functioned as informal recovery guides, where collective experience substituted for aftercare instruction.

Collectively, forum-user questions about pain represent a systemic informational failure and expose how under-explanation and institutional minimisation of hysteroscopy pain perpetuate patient disempowerment.

## Domain 3: Managing the recovery phase

Within this domain, participants’ questions reveal a search for boundaries, between normal recovery and complication, rest and exertion, fertility anxiety and reassurance. Specifically, participants wanted to know about their *restoration of health* and prepare for potential *reproductive health complexities*. Forum-users held a desire for specific knowledge that extends beyond procedural safety into personal autonomy. For example, when is uterine recovery complete? What healing benchmarks indicate readiness for conception? How may hysteroscopy affect subsequent fertility interventions?

### Return to normalcy

Post-procedural information-seeking centred on defining the boundaries of “normal” recovery: its duration, expected sensations, and timing of return to daily responsibilities. In the absence of clear clinical benchmarks, participants constructed personalised timelines, balancing soreness, fatigue and sedation effects with work and caregiving demands. They sought guidance on practical considerations including transport, mobility and activity restrictions (e.g., bathing, exercise, sexual activity and menstrual product use). Many queries focused on distinguishing expected symptoms from complications, crowd-sourcing interpretations of pain, bleeding and discharge to determine when medical review was necessary. The recurrent question of “when to seek help” highlighted the psychological burden of self-triage under conditions of incomplete aftercare instruction.

### Reproductive health complexities

Forum-users expressed uncertainty about how hysteroscopy affected menstrual cycles and reproductive timing. Questions focused on what constituted normal post-procedural recovery: when menstruation should resume, how to distinguish it from operative bleeding, and which changes warrant concern. Many struggled to differentiate expected bleeding from complications. Participants also sought guidance on when conception or fertility treatment could safely resume following hysteroscopy or coil removal. Among those undergoing assisted reproduction, uncertainty extended to the timing of IVF or embryo transfer. Fragmented communication across procedural and reproductive specialties compounded medical and emotional uncertainty, particularly within time-sensitive fertility windows.

In the absence of clear guidance, routine recovery became a period of anxious speculation, with patients crowd-sourcing timelines, pain and symptom thresholds from peers rather than clinicians. Reliance on abstract reassurance (“most women tolerate the procedure well” or “period-like cramping”) in place of specific instruction may contribute to avoidable complications, emotional distress and unscheduled healthcare use.

## Domain 4: Diagnostic outcomes and aftercare

Across this domain, the information sought out reflected a desire to know what was found during the procedure, what the findings meant, and what next steps should be taken. Yet participant queries about the *communication of diagnostic results* and *continuity of care,* exposed evident division across the diagnostic and aftercare stages. Communication breakdowns occurred not only in the content of results but in their timing, interpretation, and subsequent follow-up care.

### Communication of diagnostic results

Participant information seeking, reveals strong desire for transparency: on when and how biopsy or procedural results would be communicated and on points of contact to relieve the distress of waiting. Once results were received, participants posted online to seek clarification about their meaning. Forum-users asked about unexplained follow-up appointments and discrepancies between reported outcomes and subsequent action. Even routine communications were experienced as anxiety-inducing when insufficiently explained, leaving individuals to infer clinical meaning from insufficient detail. Instances of lost or inconclusive results deepened anxiety and powerlessness. These online queries underscore a drive for diagnostic closure where diagnostic communication is coherent and informs patient understanding and reassurance about any further treatment or discharge.

### Continuity of care

Forum-users’ searches for information after diagnosis centred on what would happen next: whether further monitoring, treatment, or follow-up contact would occur. There was a desire for continuity across the procedural and post-procedural stages, often unmet by clinical systems that discharged patients without direction. Forum-users sought explicit guidance on symptom monitoring, hormonal management, and resuming routine screening schedules, revealing how unclear pathways left them suspended between resolution and ongoing uncertainty. Many also looked for concrete points of post-procedural contact for reassurance or emotional support, asking whether hospitals provided helplines or designated staff for follow-up. Forum-users collectively attempted to reassemble disjointed care trajectories into something continuous and comprehensible.

Both themes highlight that the informational need does not end with the procedure; rather, it extends through the interpretive gaps of result communication and the ambiguities of follow-up care. This domain shows that communication breakdowns generate ongoing uncertainty that patients themselves must navigate.

### Mapping framework analysis themes to behavioural determinants

To further understand the behavioural mechanisms underpinning the identified themes, subthemes were mapped to the TDF,^55^ revealing a small number of dominant behavioural determinants of information-seeking (Table 2). Themes clustered primarily within the TDF domains of *Knowledge, Beliefs about consequences, Emotion, Environmental context and resources*, and *Behavioural regulation*. Such themes reflect efforts to manage uncertainty, anticipate risk, and navigate structural features of care rather than simple informational deficits.

**Table 2.** Mapping framework subthemes to TDF domains.

Domain	Theme	Subtheme	TDF domain(s)
Navigating Procedural Processes	Access to care	Length of waiting list	Environmental context & resources; Emotion
		Private healthcare cost	Environmental context & resources; Beliefs about consequences
		Unfamiliarity with pre-operative appointments	Knowledge; Memory, attention & decision processes
		Barriers to procedure completion	Knowledge; Environmental context & resources; Beliefs about consequences; Emotion
	Therapeutic justification	Rationale for hysteroscopy referral	Knowledge; Beliefs about consequences; Social/professional role & identity; Emotion
		Therapeutic grounds for Mirena coil	Knowledge; Beliefs about consequences; Social influences; Goals; Memory, attention & decision processes
	Preparation for operation	Logistical preparation	Knowledge; Behavioural regulation; Environmental context & resources; Skills
		Anxiety management options	Emotion; Beliefs about capabilities; Behavioural regulation; Skills
	Procedural processes	Step-by-step description	Knowledge; Memory, attention & decision processes; Emotion
		Procedural duration	Knowledge; Emotion
Anticipated Pain Trajectories	Procedural pain	Likelihood of pain occurrence	Beliefs about consequences; Emotion; Environmental context & resources; Knowledge
		Severity of procedural pain	Beliefs about consequences; Emotion; Knowledge
		Duration of procedural pain	Beliefs about consequences; Behavioural regulation; Knowledge
	Strategies for pain management	Availability of pain relief options	Knowledge; Environmental context & resources; Decision processes
		Administration of pain relief	Knowledge; Behavioural regulation
		Effectiveness of analgesia	Emotion; Beliefs about consequences; Knowledge
	Post-procedural pain	Severity of post-procedural pain	Beliefs about consequences; Emotion; Knowledge
		Duration of post-procedural pain	Beliefs about consequences; Behavioural regulation; Knowledge
Managing the Recovery Phase	Return to normalcy	Post-procedural symptoms	Knowledge; Beliefs about consequences; Emotion
		When to seek help	Knowledge; Behavioural regulation; Beliefs about consequences
		Activity restrictions during recovery	Knowledge; Behavioural regulation; Beliefs about capabilities
	Reproductive health complexities	Impact on menstrual cycles	Knowledge; Beliefs about consequences
		Guidance for fertility pathway	Knowledge; Goals; Behavioural regulation; Emotion
Diagnostic Outcomes and Aftercare	Communication of diagnostic results	Waiting times for results	Environmental context & resources; Emotion; Knowledge
		Clarity of result communication	Knowledge; Social influences; Beliefs about consequences
	Continuity of care	Provision of follow-up care	Environmental context & resources; Behavioural regulation; Social influences
		Accessibility of post-procedural support	Environmental context & resources; Social influences; Emotion

*Note.* TDF = Theoretical Domains Framework.

*Knowledge* was central, characterised by limited understanding of procedural pathways, diagnostic intent, recovery expectations and follow-up prompting attempts to construct a coherent account of hysteroscopy in the absence of anticipatory clinical explanation. *Beliefs about consequences* shaped engagement particularly in relation to pain, recovery, fertility and diagnostic outcomes, as participants sought to assess personal risk. *Emotion*, especially anxiety and fear, amplified this process, often intensified by vague communication and prolonged waiting.

*Environmental context and resources* further constrained care navigation: inconsistent scheduling, unclear referral pathways and fragmented aftercare required patients to actively interpret and manage the system. *Behavioural regulation* was evident in efforts to monitor symptoms, structure recovery and determine when to seek help. The absence of explicit behavioural thresholds reinforced reliance on peer forums as informal substitutes for aftercare guidance.

Mapping the TDF behavioural determinants identified in this study to the Behaviour Change Technique Taxonomy^56^(Table 3) provided a structure for translating participant information needs into practical, evidence-based opportunities for service improvement. This approach does not prescribe a single intervention but highlights specific techniques that could be embedded within routine communication, digital resources, and service pathways to better support informed consent, emotional preparation, and recovery.

**Table 3.** Behavioural determinants underlying participant’s information needs and corresponding behaviour change technique.

Behavioural determinant (TDF)	Example information need	Relevant BCTs (code and name)	Practical application example
Knowledge	Understanding referral rationale, procedural steps, pre-operative appointments, recovery expectations and fertility implications	4.1 Instruction on how to perform the behaviour; 5.1 Information about health consequences; 9.1 Credible source	Step-by-step procedural explainers; pre-operative videos; standardised referral explanation sheets; recovery and fertility timelines
Environmental context and resources	Navigating waiting lists, cancellations, follow-up arrangements, result communication and access to support	4.1 Instruction on how to perform the behaviour, 7.1 Prompts/cues; 12.1 Restructuring the physical environment; 12.5 Adding objects to the environment; 3.2 Social support (practical)	Transparent wait-time updates; automated follow-up scheduling; results-tracking systems; provision of aftercare helpline details
Beliefs about consequences	Anticipating pain severity, duration and variability; understanding implications of hysteroscopy and adjunct procedures (e.g. Mirena)	5.1 Information about health consequences; 9.2 Pros and cons; 5.6 Information about emotional consequences, 5.2 salience of consequences, 9.5 anticipated regret	Evidence-based pain expectation guides; decision aids comparing treatment options; clear explanation of risks, benefits and alternatives
Emotion	Managing anxiety related to pain, uncertainty, diagnostic outcomes and recovery	11.2 Reduce negative emotions; 5.6 Information about emotional consequences; 3.3 Social support (emotional)	Reassurance scripts; anxiety-reduction resources; normalisation of emotional responses; access to emotional support pre- and post-procedure; Medication offered as part of a structured anxiety-management pathway
Behavioural regulation	Knowing how to prepare, self-monitor symptoms, manage recovery and decide when to seek help	1.4 Action planning; 7.1 Prompts/cues; 1.2 Problem solving; 2.4 Self-monitoring of outcomes of behaviour	Preparation checklists; symptom diaries; clear “when to seek help” thresholds; recovery action plans delivered via digital or written formats
Goals	Aligning recovery and fertility plans with personal priorities and clinical guidance	1.1 Goal setting (outcome); 1.4 Action planning	Fertility and recovery planning tools; shared decision-making templates incorporated into follow-up consultations
Social influences	Seeking reassurance, comparison and validation from peers and clinicians, Authoritative guidance to counter informal advice	3.3 Social support (emotional); 9.1 Credible source	Clinician-endorsed information sources; signposting to appropriate support; clearer professional guidance to reduce reliance on informal advice

*Note.* TDF = Theoretical Domains Framework; BCTs = Behaviour Change Techniques.

## Discussion

### Summary of key findings

Forum-users consistently sought clarity across four main areas: referral and procedural logistics, pain and analgesia, recovery trajectories, and diagnostic result communication. Across all stages, participants requested structured, evidence-based information (timelines, benchmarks and risk estimates) to support preparation and decision-making. The dominance of basic, foundational questions suggests that core components of informed consent are not consistently conveyed. These queries concerned essential procedural understanding rather than peripheral details. From a behavioural perspective, these gaps map onto the TDF domains of deficits in knowledge, beliefs about consequences, and emotional regulation. These domains help explain why patients turn to online peer forums, not only to manage uncertainty, but also to access the social support. The peer-to-peer procedural guidance they receive, risks determining whether they attend, engage with, and complete their care.

### Comparison with existing literature

Consistent with prior research demonstrating inadequate pain communication in gynaecological care,^14^ women sought detailed and specific information about procedural and recovery pain. Participants in a qualitative investigation into women’s outpatient hysteroscopy experiences in New Zealand^57^ described pain as inadequately anticipated, pain relief options as insufficiently communicated, and information as poorly timed or inaccessible. Indeed, experimental evidence demonstrating that pain information can heighten pain perception^58^ may help explain why some clinicians report discomfort discussing analgesia.^59^ Our findings challenge the assumption that minimised disclosure is protective: participants actively sought detailed pain-related information to anticipate risk and plan recovery and reported that its absence generated anxiety. Importantly, procedural pain in hysteroscopy is clinically heterogeneous, influenced by factors including menopausal status, nulliparity, cervical stenosis, anxiety, prior pelvic pain, and clinician technique.^60,61^ The informational deficit identified in this study does not imply that pain is uniform; rather, it reflects that this clinical complexity was rarely communicated to participants in advance, leaving them to reconstruct an individualised risk picture through peer accounts.

Persistent uncertainty beyond the procedure mirrors evidence of fragmented post-procedural communication and diagnostic delays in gynaecological care.^62^ The absence of clear recovery benchmarks forced participants into self-triage,^63–65^ reinforcing feminist critiques that informational deficits reproduce gendered expectations of self-management^66,67^ and the normalisation of women’s pain.^68–70^ Reliance on online communities to reconstruct care pathways further reflects the growing substitution of digital peer support for absent institutional guidance,^71–73^ and extends it. The present findings converge with NHS survey data on patient experiences of outpatient hysteroscopy across several domains, including the centrality of pain and anxiety as informational concerns, the gap between clinical reassurance and patient experience, and the mismatch between what services consider adequate preparation and what patients report needing.^14,74^ The naturalistic forum data extend these findings by surfacing the anticipatory dimension of these concerns: what women sought to know before the procedure alongside post-procedural uncertainties around concurrent interventions, fertility guidance, and recovery self-triage.

Forum-users did not passively consume information; they actively cultivated a collective knowledge base. They sought and received reassurance and practical guidance which created an informal social support network. This support occurring amongst strangers, underlines how profoundly clinical communication failure reshapes women’s health-seeking behaviour and, by extension, their perceptions about their care.

### Implications for practice and policy

Meaningful consent requires accessible, structured information about purpose, risks and alternatives.^21^ Incomplete communication is a recognised contributor to safety incidents^21^ and, rather than being waived as a simple individual oversight, should instead be framed as a genuine institutional risk.^75^ The recurrence of basic questions across the pathway indicates that informational deficits begin at referral and persist beyond follow-up, forming an “information neglect continuum” that risks relegating consent from a meaningful empowering exchange to an insufficient procedural formality. The informational gaps identified in this study sit in direct tension with existing professional guidance. RCOG Green-top Guideline No. 59 recommends that women undergoing outpatient hysteroscopy receive individualised information about pain management options, procedural risks, and alternatives prior to consent.^16^ NICE guidance similarly mandates that patients receive clear, accessible information to support meaningful decision-making.^21^ That participants were routinely seeking this foundational information through online peer forums despite availability of NHS hysteroscopy resources, suggests a persistent implementation gap between guideline recommendation and routine clinical practice. The issue may not be one of information absence but of accessibility, adequacy, or trust: existing materials may not reach patients at the point of need, may not address the affective and experiential dimensions participants most sought, or may carry less epistemic weight than peer-generated experiential accounts.^76^

These information gaps align with behavioural techniques such as instruction, credible information provision and explanation of consequences. Recognising these gaps through a behavioural lens shifts the focus from describing problems to identifying actionable, evidence-based strategies. Concrete examples of such strategies, mapped to the specific behavioural determinants identified in this study, are provided in Table 3. These strategies can be integrated into clinical communication to improve preparedness, reduce anxiety, and influence pain and procedural outcomes. Standardised, evidence-based hysteroscopy information should therefore address referral rationale, procedural steps, pain risk, variability and analgesia, recovery thresholds and result pathways.

Digital, interactive and AI informed technologies, including telemedicine consultations and validated patient information tools, offer scalable mechanisms to deliver timely, personalised guidance across the pathway and across NHS trusts.^77–79^ Crucially, these developments must account for the virtual support infrastructure women have already built. Digital health resources should harness existing peer support mechanisms which already provide anonymity to ask difficult questions and facilitate interaction with others with lived experience. Combining digital information delivery with peer support may be more effective in improving health access, engagement, and consent quality than passive information provision. These implications align with NHS digital strategy priorities^80^ and position informational integrity as a core marker of quality and equity in women’s health services.

### Strengths and limitations

This study’s use of naturalistic online forum data captures unprompted, real-time informational needs across diverse care settings. The mean age of women undergoing outpatient hysteroscopy in UK NHS settings is approximately 47 years,^81^ which falls within Mumsnet’s most represented demographic bracket. However, the platform’s userbase may skew towards women with higher digital literacy and English-language proficiency. Socioeconomic, educational, or cultural groups less represented on Mumsnet may hold different informational priorities.^82^ Additionally, the dataset may strongly characterise negative or distressing experiences, as prior research indicates that adverse health experiences are more frequently shared online and may drive greater user engagement.^72,83^ Particularly salient or distressing accounts may disproportionately shape the nature of information-seeking observed, as emotionally vivid anecdotal narratives exert a stronger influence on health-related judgements and decision-making than statistical information.^76^ Nonetheless, online data reduce social desirability and recall bias common in interview-based research.^29,84–86^ Framework analysis enabled systematic comparison across threads and pathway stages, strengthening analytical robustness. While clinician perspectives were not directly examined, patient accounts provide meaningful insight into how informational constraints are experienced in practice. An inherent characteristic of anonymous online platforms is that individual demographic data are unavailable. However, the dataset’s scale and organic diversity partially offset concerns regarding representativeness. As the study draws exclusively on a UK-based platform, findings reflect the specific context of NHS hysteroscopy provision; experiences of women in other healthcare systems, where procedural pathways, consent practices, and educational resources may differ. However, the overlap across two methodologically distinct studies, one using clinician-mediated interviews in a single outpatient centre in New Zealand,^57^ the other analysing naturalistic peer discourse across the UK, strengthens confidence in the validity and transferability of the themes identified.

### Future research

Based on these findings, future research should aim to generate robust, stratified, quantitative predictors of outpatient hysteroscopy pain to inform patient-facing risk estimates and consent. Comparative work across gynaecological diagnostic pathways could examine whether similar informational gaps intersect with gender, deprivation and ethnicity to shape inequities. Co-designed research involving patients, clinicians and digital developers is also needed to test interactive decision aids and AI-supported information tools delivered at key pathway timepoints. Future work should also examine how virtual peer support networks function as health access infrastructure for women. Research should map how digital support shapes procedural attendance, consent quality, and care engagement, particularly among those with limited health literacy.

## Conclusion

In mapping what individuals seek to know about hysteroscopy, this study uncovered a broader pattern of systemic informational neglect within gynaecological care. Foundational questions about referral, pain and recovery appearing in public online forums signal that routine communication often fails to-at the point of need-provide the structured, evidence-based guidance patients need to understand, consent to and recover from the procedure. These informational gaps shape anticipatory anxiety, undermine trust and shift the burden of interpretation and coordination from services to patients themselves. Addressing these shortcomings requires more than incremental improvements to leaflets; it demands a deliberate commitment to informational integrity as a core component of clinical quality, safety and consent. If informational integrity is treated as a supplementary task, patients undergoing hysteroscopy will continue to bear the emotional and practical costs of such communication breakdowns. This study demonstrates that these costs are not marginal or anecdotal, but patterned, predictable and rooted in routine care processes that have thus far escaped scrutiny. That women turn to virtual peer communities is the most legible sign that informational failure has become a structural feature of gynaecological care. Digital social support has quietly become a determinant of whether women access, complete, and recover from the procedures they need.

## Supplemental Material

Supplemental Material - “*I would much rather hear the truth so I can be prepared*”: Virtual peer support, health access, and information-seeking after hysteroscopy referral Supplemental Material for “*I would much rather hear the truth so I can be prepared*”: Virtual peer support, health access, and information-seeking after hysteroscopy referral by Susanne Katharina Cromme, Richard Harrison, Katherine A. Finlay in Women’s Health

## Data Availability

The data analysed in this study are publicly accessible and can be retrieved by following the data collection procedures outlined in the Methods section.*

## References

[1] NHS . Hysteroscopy. https://www.nhs.uk/conditions/hysteroscopy/ (2024).

[2] RCOG . Outpatient hysteroscopy. https://www.rcog.org.uk/for-the-public/browse-al.l-patient-information-leaflets/outpatient-hysteroscopy/ (2022).

[3] RCOG . Left for too long: understanding the scale and impact of gynaecology waiting lists. Royal College of Obstetricians and Gynaecologists, 2022. https://rcog.shorthandstories.com/lefttoolong/index.html

[4] BSGE . RCOG report reveals devastating impact of UK gynaecology care crisis on women and NHS staff. https://www.bsge.org.uk/ (2024).

[5] RCOG . Waiting for a way forward: Voices of women and healthcare professionals at the centre of the gynaecology care crisis. Royal College of Obstetricians and Gynaecologists, 2024. https://rcog.shorthandstories.com/waiting-for-a-way-forward/

[6] Melnyk AI Pollard A Matten N , et al. No-Show Rates in a Urogynecology Clinic. UROGC 2024; 30: 314–319. 10.1097/spv.0000000000001475 PMC10947076 38484248

[7] Lindsay C Baruffati D Mackenzie M , et al. Understanding the causes of missingness in primary care: a realist review. BMC Med 2024; 22: 235. 10.1186/s12916-024-03456-2 38858690 PMC11165900

[8] Jennings B Ramis M Kynoch K , et al. Information needs of women undergoing gynaecological risk reduction surgery: Applying patient‐reported findings to improve service delivery. Aust N Z J Obstet Gynaecol 2022; 62: 286–293. 10.1111/ajo.13456 34751946

[9] Gambadauro P Navaratnarajah R Carli V . Anxiety at outpatient hysteroscopy. Gynecological Surgery 2015; 12: 189–196. 10.1007/s10397-015-0895-3 26283891 PMC4532701

[10] Akca A Yilmaz G Esmer AC , et al. Use of video-based multimedia information to reduce anxiety before office hysteroscopy. Wideochirurgia I Inne Techniki Maloinwazyjne 2020; 15: 329–336. 10.5114/wiitm.2019.89378 32489494 PMC7233155

[11] Carta G Palermo P Marinangeli F , et al. Waiting Time and Pain During Office Hysteroscopy. J Minim Invasive Gynecol 2012; 19: 360–364. 10.1016/j.jmig.2012.01.017 22387163

[12] Buzzaccarini G Alonso Pacheco L Vitagliano A , et al. Pain Management during Office Hysteroscopy: An Evidence-Based Approach. Medicina 2022; 58: 1132. 10.3390/medicina58081132 36013599 PMC9416725

[13] Law JYP . Pain relief in hysteroscopy. ng Kong J Gynaecol Obstet Midwifery 2023; 23: 46–53. 10.12809/hkjgom.23.1.08

[14] Mahmud A De Silva P Smith P , et al. Patient experiences of outpatient hysteroscopy. Eur J Obstet Gynecol Reprod Biol 2023; 288: 142–152. 10.1016/j.ejogrb.2023.07009 37531755

[15] Kokanali MK Güzel AI Özer I , et al. Pain experienced during and after office hysteroscopy with and without intracervical anesthesia. Journal of Experimental Therapeutics and Oncology 2014; 10: 243–246. 25509976

[16] De Silva PM Smith PP Cooper NAM , et al. Outpatient Hysteroscopy: (Green‐top Guideline no. 59). BJOG 2024; 131: e86–e110. 10.1111/1471-0528.17907 39160077

[17] Fahmer N Faller H Engehausen D , et al. Patients’ challenges, competencies, and perceived support in dealing with information needs – A qualitative analysis in patients with breast and gynecological cancer. Patient Educ Couns 2022; 105: 2382–2390. 10.1016/j.pec.2021.12.006 34930628

[18] Keij SM Van Duijn-Bakker N Stiggelbout AM , et al. What makes a patient ready for Shared Decision Making? A qualitative study. Patient Educ Couns 2021; 104: 571–577. 10.1016/j.pec.2020.08.031 32962880

[19] Temmerman M Khosla R Say L . Sexual and reproductive health and rights: a global development, health, and human rights priority. Lancet 2014; 384: e30–e31. 10.1016/S0140-6736(14)61190-9 25043387

[20] World Health Organisation . Progress reports: report by the Director-General. In: Seventy-eighth World Health Assembly, Geneva, 2025 Report No.: A78/34.(provisional agenda item 28.1.

[21] NICE . Clinical guideline: Patient experience in adult NHS services: improving the experience of care for people using adult NHS services. Reference number: CG138. National Institute for Health and Care Excellence. https://www.nice.org.uk/guidance/cg138/chapter/Recommendations (17 June 2021 34260162

[22] Fernandez S Short SC Boele F . Glioblastoma Patient and Caregiver Perspectives of Treatment Side-Effects and Information Provision. J Patient Exp 2025; 12: 23743735251331770. 10.1177/23743735251331770 40290735 PMC12032473

[23] Low M Burgess LC Wainwright TW . Patient Information Leaflets for Lumbar Spine Surgery: A Missed Opportunity. J Patient Exp 2020; 7: 1403–1409. 10.1177/2374373519897176 33457594 PMC7786772

[24] McCartney M . Patient information leaflets: ‘a stupid system. Brit Med J 2013; 347: 4748. 10.1136/bmj.f4748 23900316

[25] Department of Health and Social Care . The Renewed Women’s Health Strategy for England London, 2026; CP 1558. https://www.gov.uk/government/publications/renewed-womens-health-strategy-for-england

[26] Bringley J Sundaram P Avis E , et al. Effect of age on U.S. gynecologic patients’ use of social media for women’s health information. Patient Educ Couns 2023; 114: 107809. 10.1016/j.pec.2023.107809 37244132

[27] Broussard K Becker A . Self-removal of long-acting reversible contraception: A content analysis of YouTube videos. Contraception 2021; 104: 654–658. 10.1016/j.contraception.2021.08.002 34400154 PMC8592268

[28] Gunnarsson L Wemrell M . On the verge between the scientific and the alternative: Swedish women’s claims about systemic side effects of the copper intrauterine device. Public Underst Sci 2023; 32: 175–189. 10.1177/09636625221107505 35900002 PMC9900186

[29] Taghinejadi N van der Westhuizen HM Ayomoh FI , et al. Pain experiences during intrauterine device procedures: a thematic analysis of tweets. BMJ Sex Reprod Health 2024; 50: 271–277. 10.1136/bmjsrh-2023-202011 PMC11503099 38862196

[30] Cromme SK Harrison R Finlay KA . From pain gaslighting to gender biases in women’s accounts of hysteroscopy: A qualitative reflexive thematic analysis. Womens Health (Lond Engl) 2026; 22: 17455057261440884. 10.1177/17455057261440884 42009054 PMC13110283

[31] Croucher L Mertan E Shafran R , et al. The Use of Mumsnet by Parents of Young People With Mental Health Needs: Qualitative Investigation. JMIR Ment Health 2020; 7: e18271. 10.2196/18271 32880583 PMC7499161

[32] Mumsnet . Advertising With Mumsnet. https://www.mumsnet.com/i/advertising (accessed 20 March 2023).

[33] Pedersen S . “It’s what the suffragettes would have wanted”: the construction of the suffragists and suffragettes on Mumsnet. Feminist Media Studies 2023; 23: 1543–1558. 10.1080/14680777.2022.2032788

[34] Pedersen S . Practical, everyday feminism: mothers, politicians, and Mumsnet. Women’s History Review 2021; 30: 509–519. 10.1080/09612025.2020.1860281

[35] Ehrstein Y . “Facilitating wife” and “feckless manchild”: Working mothers’ talk about divisions of care on Mumsnet. Feminism & Psychology 2022; 32: 394–412. 10.1177/09593535221094260

[36] Bailey L . The virtual mother: Mumsnet and the emergence of new forms of ‘good mothering’ online. Discourse & Communication 2023; 17: 40–56. 10.1177/17504813221123663

[37] Hall M Van Hooff J . Otherland: accounts of ordinary childless/freeness on Mumsnet. Families, Relationships and Societies 2024; 13: 140–156. 10.1332/204674321x16738753257034

[38] Mayoh J McDonald K Luce A . Listening to women’s personal stories about suicide: an online thematic analysis of the discourse on UK parenting forum Mumsnet. Feminist Media Studies 2024; 25: 1–18. 10.1080/14680777.2024.2375390

[39] Von Benzon N Hickman-Dunne J Whittle R . ‘My doctor just called me a good girl and I died a bit inside’: From everyday misogyny to obstetric violence in UK fertility and maternity services. Social Science & Medicine 2024; 344: 116614. 10.1016/j.socscimed.2024.116614 38308962

[40] Klejnotowska AE Skea ZC Cotton SC , et al. Exploring Opinions and Concerns about the Human Papillomavirus Vaccine on a United Kingdom Online Discussion Forum. Journal of Colposcopy and Lower Genital Tract Pathology 2023; 1: 117–123. 10.4103/jclgtp.jclgtp_20_23

[41] British Psychological Society . Ethics guidelines for internet-mediated research. British Psychological Society.

[42] National Guideline Alliance Great Britain . Heavy menstrual bleeding: assessment and management. National Institute for Health and Care Excellence, 2018. 29634173

[43] R Core Team . R: A language and environment for statistical computing. https://www.R-project.org/ (2021).

[44] Wickham H . rvest: Easily Harvest (Scrape) Web Pages. R package version 1.0.4. https://github.com/tidyverse (2024).

[45] Wickham H Girlich M Ruiz E . dbplyr: A ‘dplyr’ Back End for Databases. R package version 2.5.0. 2024 10.32614/CRAN.package.dbplyr

[46] Wickham H Girlich M Ruiz E . tidyr: Tidy Messy Data. R package version. 2024 1.3.1 https://CRAN.R-project.org/package=tidyr

[47] VERBI Software . MAXQDA 2021, Version 2022 [computer software], 2021. Berlin https://www.maxqda.com/

[48] Ritchie J Spencer L . Qualitative Data Analysis for Applied Policy Research. The Qualitative Researcher’s Companion. 2455 Teller Road, Thousand Oaks California 91320 United States of America. Sage Publications, Inc, pp. 305–329.

[49] Tong A Sainsbury P Craig J . Consolidated criteria for reporting qualitative research (COREQ): a 32-item checklist for interviews and focus groups. Int J Qual Health Care 2007; 19: 349–357. 10.1093/intqhc/mzm042 17872937

[50] Gale NK Heath G Cameron E , et al. Using the framework method for the analysis of qualitative data in multi-disciplinary health research. BMC Med Res Methodol 2013; 13: 117. 10.1186/1471-2288-13-117 24047204 PMC3848812

[51] Thomas DR . A General Inductive Approach for Analyzing Qualitative Evaluation Data. Am J Eval 2006; 27: 237–246. 10.1177/1098214005283748

[52] Braun V Clarke V . To saturate or not to saturate? Questioning data saturation as a useful concept for thematic analysis and sample-size rationales. Qualitative Research in Sport, Exercise and Health 2021; 13: 201–216. 10.1080/2159676x.2019.1704846

[53] Braun V Clarke V . Thematic Analysis: A Practical Guide. 2022 London: SAGE Publications 10.53841/bpsqmip.2022.1.33.46

[54] Braun V Clarke V . Is thematic analysis used well in health psychology? A critical review of published research, with recommendations for quality practice and reporting. Health Psychology Review 2023; 0: 1–24. 10.1080/17437199.2022.2161594 36656762

[55] Cane J O’Connor D Michie S . Validation of the theoretical domains framework for use in behaviour change and implementation research. Implementation Sci 2012; 7: 37. 10.1186/1748-5908-7-37 PMC3483008 22530986

[56] Michie S Richardson M Johnston M , et al. The Behavior Change Technique Taxonomy (v1) of 93 Hierarchically Clustered Techniques: Building an International Consensus for the Reporting of Behavior Change Interventions. ann behav med 2013; 46: 81–95. 10.1007/s12160-013-9486-6 23512568

[57] Nunnerley J Sheppard L Springer R , et al. Should It Hurt? Experiences of Out-Patient Clinic-Based Hysteroscopy. Australian and New Zealand Journal of Obstetrics and Gynaecology 2026; 66: e70089. 10.1111/ajo.70089 41416620

[58] Swannell ER Brown CA Jones AKP , et al. Some Words Hurt More Than Others: Semantic Activation of Pain Concepts in Memory and Subsequent Experiences of Pain. J Pain 2016; 17: 336–349. 10.1016/j.jpain.2015.11.004 26681115

[59] Hillard PJA . Learning to Communicate With Patients About Potentially Painful Gynecologic Procedures. AMA J Ethics 2025; 27: E91–E97. 39899821 10.1001/amajethics.2025.91

[60] Anggraeni TD Davis GS Kusuma F , et al. Association Between Menstrual Pain and Procedural Pain During Office Hysteroscopy: A 10-Year Cohort Study. Pain Research and Management 2026; 2026: 8879788. 10.1155/prm/8879788 41737893 PMC12927967

[61] Vicentin RTI Haddad RF de Figueiredo JS , et al. Factors Associated with Pain Levels During Office Hysteroscopy: A Cross-Sectional Study. Women 2025; 5: 32. 10.3390/women5030032

[62] Booth K Beaver K Kitchener H , et al. Women’s experiences of information, psychological distress and worry after treatment for gynaecological cancer. Patient Educ Couns 2005; 56: 225–232. 10.1016/j.pec.2004.02.016 15653253

[63] Huband H McGarragle KM Hare CJ , et al. Gynecologic cancer screening among women with Lynch syndrome: Information and healthcare access needs. Patient Educ Couns 2025; 131: 108576. 10.1016/j.pec.2024.108576 39644530

[64] Spencer RA Singh Punia H . A scoping review of communication tools applicable to patients and their primary care providers after discharge from hospital. Patient Educ Couns 2021; 104: 1681–1703. 10.1016/j.pec.2020.12.010 33446366

[65] Abernethy A Adams L Barrett M , et al. The Promise of Digital Health: Then, Now, and the Future. NAM Perspectives 2022; 6: 1–24. 10.31478/202206e PMC9499383 36177208

[66] Klostermann J Funk L Symonds-Brown H , et al. The Problems with Care: A Feminist Care Scholar Retrospective. Societies 2022; 12: 52. 10.3390/soc12020052

[67] Llubes-Arrià L Briones-Vozmediano E Mateos JT , et al. Gender differences in self-management activation among patients with multiple chronic diseases: a qualitative study. Arch Public Health 2025; 83: 200. 10.1186/s13690-025-01686-1 40745663 PMC12315370

[68] Bayer L McWilliams EA . How Should Intensity and Duration of Pain Inform Standard of Care for Pain Management in Non-Labor and Delivery OB/GYN Procedures? AMA J Ethics 2025; 27: E98–E103. 39899822 10.1001/amajethics.2025.98

[69] Purcell C Greenfield M . Experiences of Pain in Sexual &Reproductive Health Procedures. Policy Brief, 2025. https://oro.open.ac.uk/105011/1/OUPainfulProceduresPolicyBriefJune2025.pdf

[70] Robstad N Paulsen A Vistad I , et al. Experiences of pain communication in endometriosis: A meta‐synthesis. Acta Obstet Gynecol Scand 2025; 104: 39–54. 10.1111/aogs.14995 39440568 PMC11683542

[71] Davison KP Pennebaker JW Dickerson SS . Who talks? The social psychology of illness support groups. American Psychologist 2000; 55: 205–217. 10717968

[72] Gilbert E Perz J Ussher JM . Talking about sex with health professionals: the experience of people with cancer and their partners. 2016 Eur J Cancer Care (Engl) 25(2): 280–293. 10.1111/ecc.12216 25040442

[73] Lupton D Maslen S . How Women Use Digital Technologies for Health: Qualitative Interview and Focus Group Study. J Med Internet Res 2019; 21: e11481. 10.2196/11481 30681963 PMC6367665

[74] Mahmud A Smith P Clark TJ . Benchmarking services in outpatient hysteroscopy (OPH): A quality improvement project. European Journal of Obstetrics & Gynecology and Reproductive Biology 2021; 259: 211–221. 10.1016/j.ejogrb.2021.01.028 33573857

[75] Cream J Wellings D Wenzel L , et al. Lost in the system: the need for better admin. The Kings Fund, 2025. https://www.kingsfund.org.uk/insight-and-analysis/long-reads/lost-in-system-need-for-better-admin

[76] Freling TH Yang Z Saini R , et al. When poignant stories outweigh cold hard facts: A meta-analysis of the anecdotal bias. Organizational Behavior and Human Decision Processes 2020; 160: 51–67. 10.1016/j.obhdp.2020.01.006

[77] Algeri P Adorni M Bergamelli S , et al. Telemedicine in support of post‐surgical follow‐up: A useful and accessible method, appreciated by the patient, to improve management after gynecological surgery. Int J Gynaecol Obstet 2025; 172: 271–278. 10.1002/ijgo.70350 40616340

[78] Mabillard V Demartines N Joliat GR . How Can Reasoned Transparency Enhance Co-Creation in Healthcare and Remedy the Pitfalls of Digitization in Doctor-Patient Relationships? Int J Health Policy Manag 2021; 11: 1986–1990. 10.34172/ijhpm.2020.263 33590744 PMC9808292

[79] Maleki Varnosfaderani S Forouzanfar M . The Role of AI in Hospitals and Clinics: Transforming Healthcare in the 21st Century. Bioengineering 2024; 11: 337. 10.3390/bioengineering11040337 38671759 PMC11047988

[80] Department of Health and Social Care . Fit for the future: 10 Year Health Plan for England. DHSC, 2025. Policy Paper. https://www.gov.uk/government/publications/10-year-health-plan-for-england-fit-for-the-future/fit-for-the-future-10-year-health-plan-for-england-executive-summary

[81] Gulumser C Narvekar N Pathak M , et al. See-and-treat outpatient hysteroscopy: an analysis of 1109 examinations. Reproductive biomedicine online 2010; 20(3): 423–429. 10.1016/j.rbmo.2009.11.024 20096633

[82] Bond CS Ahmed OH Hind M . Implications for Research Methods When Conducting Studies With the Users of Online Health Communities. Comput Inform Nurs 2014; 32: 101–104. 10.1097/CIN.0000000000000049 24642856

[83] Robertson CE Pröllochs N Schwarzenegger K , et al. Negativity drives online news consumption. Nat Hum Behav 2023; 7: 812–822. 10.1038/s41562-023-01538-4 36928780 PMC10202797

[84] Allen RH Song S Weir GM , et al. “Stop Gaslighting Your Patients”: A Quantitative and Qualitative Analysis of User Experiences of IUDs on TikTok. J Pediatr Adolesc Gynecol 2025; 38: 498–503. 10.1016/j.jpag.2025.02.003 39938712

[85] Merz AA Gutiérrez-Sacristán A Bartz D , et al. Population attitudes toward contraceptive methods over time on a social media platform. Am J Obstet Gynecol 2021; 224: 597.e1–597.e14. 10.1016/j.ajog.2020.11.042 33309562

[86] Wu J Trahair E Happ M , et al. TikTok, #IUD, and User Experience With Intrauterine Devices Reported on Social Media. Obstet Gynecol 2023; 141: 215–217. 10.1097/aog.0000000000005027 36473194 PMC9892286

